# Pre-Existing Atrial Fibrillation in Hospitalized Patients with COVID-19: Insights from the CARDIO COVID 19–20 Registry

**DOI:** 10.3390/jcdd11070210

**Published:** 2024-07-04

**Authors:** Wikler Bernal Torres, Juan Pablo Arango-Ibanez, Juan Manuel Montero Echeverri, Santiago Posso Marín, Armando Alvarado, Andrés Ulate, Paola Oliver, Ivan Criollo, Wilbert German Yabar Galindo, Sylvia Sandoval, William Millán Orozco, Fernando Verdugo Thomas, Franco Appiani Florit, Andrés Buitrago, Alejandra Ines Christen, Igor Morr, Luiz Carlos Santana Passos, Marlon Aguirre, Roger Martín Correa, Hoover O. León-Giraldo, Andrea Alejandra Arteaga-Tobar, Juan Esteban Gómez-Mesa

**Affiliations:** 1 Fundación Valle del Lili, Departamento de Cardiología, Cali 760032, Colombia; wikler.bernal@fvl.org.co; 2 Facultad de Ciencias de la Salud, Universidad Icesi, Cali 760032, Colombia; santiago.posso@fvl.org.co (S.P.M.); hoover.leon.gi@fvl.org.co (H.O.L.-G.); andrea.arteaga@fvl.org.co (A.A.A.-T.); 3 Fundación Valle del Lili, Centro de Investigaciones Clínicas, Cali 760032, Colombia; juan.arango.ib@fvl.org.co (J.P.A.-I.);; 4 Hospital Especializado de Villa Nueva, Villa Nueva 01064, Guatemala; santostemplarios@gmail.com; 5 Hospital México, San Jose 10103, Costa Rica; andresulre77@gmail.com; 6 Hospital Nacional Arzobispo Loayza, Lima 15082, Peru; paolaoliver.re@gmail.com; 7 Hospital Regional Arica, Arica y Parinacota 1000875, Chile; icll53@hotmail.com; 8 Hospital Nacional Guillermo Almenara Irigoyen, Lima, Peru; yabar.galindo@gmail.com; 9 Centro de Atención Temporal Quito Solidario, Quito, Ecuador; sxsc_@hotmail.com; 10 Hospital San José de Buga, Buga, Colombia; wmillan1984@gmail.com; 11 Hospital Militar, Santiago de Chile, Chile; fdoverdugo@gmail.com; 12 Hospital Dirección Previsional de Carabineros, Santiago de Chile, Chile; fappiani@gmail.com; 13 Servicio de Cardiología, Fundación Santa Fe de Bogotá, Bogotá 110111, Colombia; 14 Hospital Presidente Perón, Buenos Aires B1872AWK, Argentina; 15 Comité de Cardiología Tropical—Sociedad Venezolana de Cardiología, Caracas 1011, Venezuela; igormorr@hotmail.com; 16 Hospital Ana Nery—HAN/SESAB, Salvador de Bahía 40301-155, Brazil; lcpassos@ufba.br; 17 Hospital Metropolitano, Quito 170521, Ecuador; dr.marlonaguirre1256@hotmail.com; 18 Hospital Nacional Alberto Sabogal Sologuren, Bellavista 07011, Peru

**Keywords:** atrial fibrillation, COVID-19, mortality, risk factors, arrhythmia

## Abstract

Pre-existing (chronic) atrial fibrillation (AF) has been identified as a risk factor for cardiovascular complications and mortality in patients with COVID-19; however, evidence in Latin America (LATAM) is scarce. This prospective and multicenter study from the CARDIO COVID 19–20 database includes hospitalized adults with COVID-19 from 14 countries in LATAM. A parsimonious logistic regression model was used to identify the main factors associated with mortality in a simulated case-control setting comparing patients with a history of AF to those without. In total, 3260 patients were included, of which 115 had AF. The AF group was older, had a higher prevalence of comorbidities, and had greater use of cardiovascular medications. In the model, AF, chronic kidney disease, and a respiratory rate > 25 at admission were associated with higher in-hospital mortality. The use of corticosteroids did not reach statistical significance; however, an effect was seen through the confidence interval. Thus, pre-existing AF increases mortality risk irrespective of other concomitant factors. Chronic kidney disease and a high respiratory rate at admission are also key factors for in-hospital mortality. These findings highlight the importance of comorbidities and regional characteristics in COVID-19 outcomes, in this instance, enhancing the evidence for patients from LATAM.

## 1. Introduction

The COVID-19 pandemic has caused a major global crisis since its declaration in March 2020 [1], posing numerous challenges to the medical and scientific community. COVID-19 impacts the cardiovascular system significantly, leading to complications in both the acute phase and the long-term course of the disease [2]. Considering these implications, various cardiovascular conditions have been explored to identify risk factors linked to poorer outcomes. Among these, atrial fibrillation (AF) has been associated with a higher rate of cardiovascular complications, intensive care unit (ICU) admission, and in-hospital mortality [3,4]. AF is the most common cardiac arrhythmia in clinical practice and among patients with COVID-19. Moreover, it is associated with other known important comorbidities for poor COVID-19 outcomes, such as high blood pressure, heart failure, and diabetes mellitus [5,6,7]. 

AF is not just a risk factor for adverse outcomes in COVID-19 patients; it also arises as a complication of the infection due to the prothrombotic and pro-inflammatory state seen in these patients. Several mechanisms are proposed to explain the poorer outcomes in patients with pre-existing (chronic) AF and the emergence of new AF. These mechanisms encompass an inflammatory and prothrombotic state, reduced angiotensin-converting enzyme II activity (contributing to increased cardiac remodeling), and endothelial dysfunction, among others [7]. These mechanisms, closely associated with the cardiovascular system, elucidate the increased risk of significant adverse cardiovascular events and poorer outcomes in patients with AF during COVID-19 infection.

COVID-19 has demonstrated significant geographical and ethnic disparities. In particular, it has deeply impacted Latin America’s (LATAM) economy and public health [8]. AF also places a significant disease burden on LATAM due to inadequate treatment and limited epidemiological research [9,10]. Given the increased mortality risk associated with pre-existing AF in COVID-19 patients, it is imperative to adopt comprehensive cardiovascular care strategies, tailored to the unique challenges posed by the SARS-CoV-2 infection, especially in regions where poorer outcomes have been reported [11]. This study evaluates the characteristics of hospitalized COVID-19 patients with pre-existing (chronic) AF and analyzes factors associated with in-hospital mortality. This issue continues to be important as the Americas have seen a decrease in vaccination coverage and increased vaccine hesitancy in the post-pandemic period [12].

## 2. Materials and Methods

### 2.1. Design and Study Population

This is a cohort study conducted using the CARDIO COVID 19–20 database, which is a prospective, multicentric study that included patients with COVID-19 from 44 hospitals across 14 Latin American countries (Argentina, Brazil, Chile, Colombia, Costa Rica, Dominican Republic, Ecuador, El Salvador, Guatemala, Mexico, Panama, Paraguay, Peru, and Venezuela) [13,14]. Inclusion criteria for this study include the following:

- Adult patients (18 years or older);
- A confirmed serological diagnosis of COVID-19;
- Patients admitted and hospitalized, or those who died within 24 h of admission.

Patients meeting the inclusion criteria were enrolled in the study from 1 June 2020 to 30 June 2021. The Consejo Interamericano de Falla Cardíaca e Hipertensión Pulmonar (CIFACAH) of the Sociedad Interamericana de Cardiología (SIAC) organized and supervised the database [13].

### 2.2. Definitions

This study focuses on patients with a history of AF before hospital admission, often referred to as chronic or pre-existing AF [15,16]. In this context, AF will denote pre-existing AF, as opposed to new-onset or de novo AF, which is diagnosed after admission [17]. We specify each time a new-onset AF is discussed.

### 2.3. Statistical Analysis

An exploratory analysis was conducted to verify the quality of the data, and the process of cleaning and filling in the information was completed. Qualitative variables were described with frequencies and percentages, and quantitative variables with central tendency and dispersion measures. To evaluate the assumption of normal distribution, the Shapiro–Wilk test was used.

The primary outcome was in-hospital death. Bivariate and multiple logistic regression were used to evaluate associations between clinical variables (demographics, comorbidities, cardiovascular treatment, clinical findings, and cardiovascular complications). The results were presented as odds ratios (OR) with their respective 95% confidence intervals.

To perform a logistic regression model, variables were selected based on their *p*-values (<0.25) in a preliminary analysis to include potential predictors of hospital mortality among COVID-19 patients. This follows the guidelines proposed by Hosmer and Lemeshow [18]. We used a nested case-control approach with estimated parameters (simulating controls) to address the significant imbalance between the number of patients with AF (113 patients) and those without (3145 patients). We simulated a 2:1 case-control study by randomly selecting two patients without AF for each patient with AF, creating 1000 comparison groups. This approach aimed to balance the groups and enhance the accuracy of the association analysis. A binary logistic regression model was adjusted for each of these simulated datasets, generating 1000 sets of parameter estimates. The final model parameters were determined by averaging these estimates, with confidence intervals constructed from the 2.5th and 97.5th percentiles of the distribution of these estimates. Patients with new-onset AF were not included in the statistical analysis.

### 2.4. Ethical Considerations

This study was approved by the Comité de Ética (institutional review board) of Fundación Valle del Lili (approval #1835) and by the ethics committee of each participating institution. It adheres to the ethical guidelines outlined in the Declaration of Helsinki. No informed consent was required as it is an observational study with data from electronic health records.

## 3. Results

The CARDIO COVID 19–20 registry enrolled 3260 patients. Patients with a history of AF and COVID-19 (*n* = 115) presented differential demographic characteristics and comorbidities (Table 1) compared to those without AF (*n* = 3145). Patients with AF were significantly older compared to those without AF (median: 72.0 vs. 61.0 years, respectively; *p* < 0.001). The pooled in-hospital mortality rate was 25.5%. Notably, 46 patients (40%) with AF died, compared to 785 (25%) without AF (*p* < 0.001). No patients in this registry were vaccinated against COVID-19.

The prevalence of most comorbidities was also higher in patients with AF, including hypertension (HTN) (70.4% vs. 48.2%, *p* < 0.001), dyslipidemia (30.4% vs. 13.2%, *p* < 0.001), chronic kidney disease (CKD) (24.3% vs. 7.7%, *p* < 0.001), coronary artery disease (CAD) (23.5% vs. 6.9%, *p* < 0.001), heart failure (HF) (43.5% vs. 4.2%, *p* < 0.001), stroke (15.7% vs. 2.7%, *p* < 0.001), and the use of cardiac implantable devices (13% vs. 1.3%, *p* < 0.001).

Table 2 demonstrates the clinical characteristics of both groups. Patients with AF had a higher prevalence of using cardiovascular medications such as angiotensin-converting enzyme inhibitors (ACEIs) (24.3% vs. 10.5%, *p* < 0.001), angiotensin II receptor blockers (ARBs) (37.4% vs. 24.2%, *p* = 0.002), anticoagulants (66.1% vs. 2.4%, *p* < 0.001), and diuretics (45.2% vs. 9.3%, *p* < 0.001). Both groups had similar in-hospital use of corticosteroids and mechanical ventilation but differed regarding anticoagulation (73.9% vs. 37.3%), inotropic (28.7% vs. 9.6%), and vasopressor (36.5% vs. 27.3%).

Table 3 represents the echocardiographic findings of patients with AF. From the 51 available, the median left ventricular ejection fraction was 45 (IQR: 36.5, 58.5). Systolic and right ventricular dysfunction were present in 47% and 33% of these patients, respectively. The most common severe valve disease was mitral regurgitation, present in 9.8% of patients with AF and echocardiography.

### Logistic Regression Analysis

In the bivariate logistic regression analysis (Table 4), multiple variables were included: HTN, diabetes mellitus, CKD, CAD, stroke, smoking, use of beta-blockers, respiratory rate (RR), pulmonary thromboembolism, acute coronary syndrome, use of corticosteroids, and invasive mechanical ventilation. In the regression model, the inclusion of the variable ‘invasive mechanical ventilation’ was evaluated, given its much higher estimate than other variables in the bivariate analysis (OR = 11.22, *p* = 0.03).

**Figure 1:**
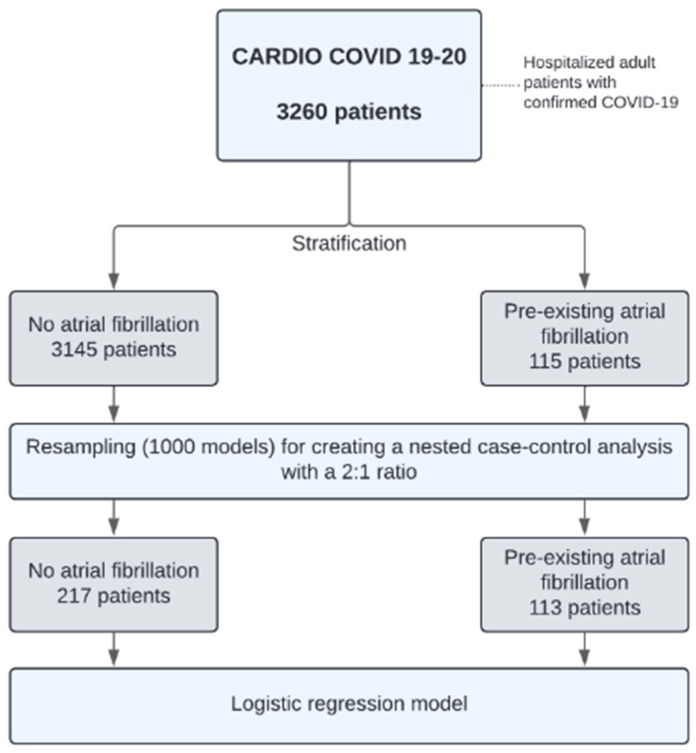
Flowchart of the case-control study simulation for logistic regression modeling.

**Figure 2:**
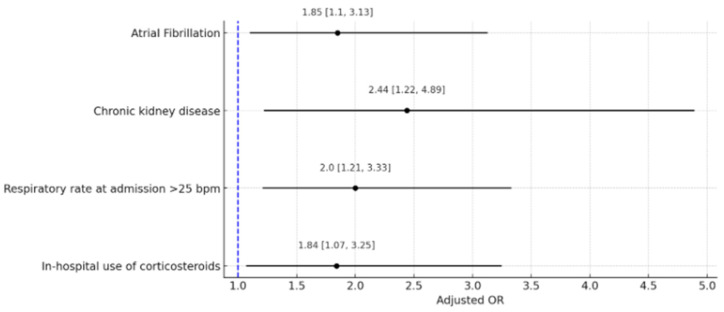
Forrest plot of clinical variables associated with in-hospital mortality. This graph displays a Forest plot of factors associated with in-hospital mortality from COVID-19, obtained through a regression model. The adjusted odds ratios (OR) for each factor are shown above the corresponding lines, with confidence intervals presented within brackets. Bpm: breaths per minute.

Figure 1 illustrates the process for simulating a case-control study to optimize our statistical evaluation of AF. The final model simulated a 2:1 case-cohort study, including 113 patients with AF and 217 controls (statistically representative of patients without AF), representing a subcohort sample of 6.8%. The adjusted logistic regression model revealed that independent of other variables in the model, having a history of AF was associated with an increased risk of in-hospital mortality (adjusted OR 1.85, 95% CI 1.10–3.13, *p* = 0.044). Other variables included CKD (adjusted OR 2.44, 95% CI 1.22–4.89, *p* = 0.03), respiratory rate > 25 breaths per minute (bpm) (adjusted OR 2.00, 95% CI 1.21–3.33, *p* = 0.032), and the use of corticosteroids (adjusted OR 1.84, 95% CI 1.07–3.25, *p* = 0.08) (Table 5 and Figure 2). These variables represent the most important factors for mortality in the case-control simulation.

## 4. Discussion

SARS-CoV-2 infection and its cardiovascular implications have been the research subject in multiple studies. Nevertheless, some aspects still need more investigation, particularly in areas with limited literature on COVID-19, such as LATAM [19,20]. This secondary analysis of the CARDIO COVID-19–20 registry explored the association of pre-existing AF with mortality as the main clinical outcome in hospitalized patients diagnosed with COVID-19. It was found that, regardless of the presence of other risk factors, AF is significantly associated with an increased risk of mortality.

**Atrial fibrillation:** In a recent study including 1241 patients with COVID-19, pre-existing AF was present in 7.6% of them. Statistical significance was found for new-onset AF, but not for pre-existing AF (OR: 1.13, 95% CI 0.57–2.21, *p* = 0.732) after adjusting for race and preadmission CHA2DS2-VASC [21]. In a retrospective observational study of 7713 patients with COVID-19 in South Korea, only 0.8% of the patients had a history of AF. However, this group exhibited a higher prevalence of severe complications and a strong association with mortality (OR 2.09, 95% CI 1.01–4.31, *p* = 0.048) [19]. A meta-analysis of 14 studies revealed that the presence of AF increased mortality from any cause by up to four times in patients with COVID-19. It is important to note that in this meta-analysis, the heterogeneity of the studies was high, and a risk association was observed only during the univariate analysis [22]. In our study, the prevalence of AF differs from the previously exposed studies, which population differences may explain. Our findings document that the risk association between AF and COVID-19 infection is maintained in both univariate and multivariate analyses, which suggest a consistently significant effect after adjustment to other variables.

In the HOPE registry, including 24 centers in Spain, 4.5% of the patients had a history of AF [23]. Interestingly, our population presents similar characteristics compared to the one from the mentioned study. Patients with AF tended to be older, which also occurred in our study. The majority of patients in the group of AF were also male, as seen in our results. Moreover, mortality was also higher in the group with AF (hazard ratio 1.2). These results underscore the parallels in patient characteristics between the individuals in this study and those in our research. 

The SEMI-COVID19 study [24], including 16,461 Spanish patients, revealed that 11% of them had FA. This study found that patients with AF had a higher prevalence of other comorbidities, such as diabetes mellitus and hypertension, a finding that is also present in our data. The study also identified an association between AF and mortality, although in a univariate analysis. An adjusted analysis showed that factors such as advanced age, a higher Charlson Comorbidity Index, elevated serum creatinine levels, and increased C-reactive protein were independently linked to mortality in patients with AF. However, the individual impact of each of these variables on mortality was modest.

In our study, we found that patients with pre-existing AF had a significantly higher prevalence of cardiometabolic comorbidities and a higher median age. These are well-documented risk factors contributing to increased mortality [6]. This observation implies that the elevated mortality risk in patients with AF may not be solely attributable to the AF itself but rather to the cumulative effect of multiple concurrent risk factors. However, after adjusting for other factors in our model, AF consistently presented as a factor associated with mortality.

Given the biological plausibility, temporal association, consistency with literature findings, and control for confounding factors, our study underscores AF as a significant risk factor for mortality among Latin American patients hospitalized with COVID-19.

**Chronic kidney disease:** Our regression model revealed that CKD is a risk factor for in-hospital mortality. Furthermore, a meta-analysis encompassing 382,407 patients with CKD indicated a higher incidence of infection and mortality [25]. Another meta-analysis, which included 1342 patients with CKD, reported an increased risk of disease severity [26]. A meta-analysis revealed a significantly higher risk of mortality in patients with chronic kidney disease (CKD), with a substantial effect size (OR 5.81, 95% CI 3.78–8.94, *p* < 0.00001) [27]. 

One study indicated that patients with both CKD and AF had a higher mortality risk compared to those without CKD; this was specifically significant for new-onset AF (HR 1.732, 95% CI 1.2–2.3) [15]. We observed a similar effect size, suggesting that CKD is associated with mortality in patients with both new-onset and pre-existing AF (our cohort). The case of CKD as a factor for mortality in patients with AF and COVID-19 is likely attributable to its substantial effect size on mortality, as suggested by the research previously discussed.

**Respiratory rate at admission:** A respiratory rate > 25 bpm at admission was found to be associated with mortality in patients with AF. Chatterjee et al. conducted a study including 1095 patients that identified an association between tachypnea (>20 breaths per minute) and mortality in hospitalized COVID-19 patients (RR 1.79, 95% CI 1.18–2.70, *p* < 0.001). The risk of death increased as the respiratory rate increased, reaching an RR of 1.72 for respiratory rates between 25 and 28 breaths per minute, and an RR of 3.20 for respiratory rates greater than 30 breaths per minute [28]. Another study found that the median respiratory rate for non-survivors was 24, an observation similar to our findings [29]. This finding was also reported by a meta-analysis [30]. The association between an elevated respiratory rate and increased mortality in COVID-19 is expected, considering its strong association with dyspnea and severe disease, both established risk factors for mortality [31].

**Corticosteroids:** Corticosteroids have shown promising effects for treating COVID-19 since the beginning of the pandemic [32]; however, subsequent studies have demonstrated mixed results. One meta-analysis including 7907 patients from randomized controlled trials showed a pooled reduction in all-cause mortality (RR 0.88, 95% CI 0.82–0.95, *p* < 0.002), but a greater effect was seen in patients with severe COVID-19 (RR 0.77, 95% CI 0.68–0.88, *p* < 0.0001) [33]. Another meta-analysis including 43 studies and 77,426 patients who received corticosteroid therapy showed a significant increase in mortality when compared to a placebo (RR 2.173, 95% CI 2.069–2.282), especially in those receiving methylprednisolone [34]. The most recent meta-analysis investigating this question compared methylprednisolone and dexamethasone in patients with severe COVID-19 and found that methylprednisolone was associated with reduced short-term mortality [35]. 

In our cohort of patients, corticosteroid use showed no statistical significance, but an effect was observed (aOR 1.84, 95% CI 1.07-3.25, *p* < 0.08). Statistical variability may prevent the *p*-value from reaching the conventional significance threshold of 0.05, despite the confidence interval not including the unit (zero), which could indicate a significant effect [36]. Additionally, a non-linear relationship may exist between corticosteroid use and mortality risk, indicating disproportionate variable changes. This variability might stem from the employment of different corticosteroid types, each with varying effects. The incorporation of corticosteroid use in our models was important as this model outperformed other models (in terms of area under the curve) lacking this variable. Given these findings, further research is warranted to elucidate corticosteroids’ impact on general patient mortality, particularly in those with AF.

### Strengths and Limitations

The study’s most notable strength lies in its multinational and relatively large sample size, encompassing 3260 patients across 44 hospitals in 14 countries in LATAM. Additionally, the prospective, multicentric design of the CARDIO COVID 19–20 registry ensures comprehensive and real-time data collection. The logistic regression analysis and the approach to addressing sample size imbalances further underscore the study’s methodological rigor.

One limitation of the study is the use of a model that, although it simplifies the analysis by avoiding problems such as multicollinearity, may omit important variables. This strategy, while increasing stability and facilitating the interpretation of the model, could exclude relevant factors that provide additional insights into mortality in this population. Our findings should be interpreted with caution. While they highlight the primary factors associated with mortality, they do not suggest that these are the only factors and that they are representative of the constructed model.

The statistical analysis conducted suggests that the identified risk factors are primarily indicative of the differences between a group with AF and a comparative group without it. The notable imbalance between the patient groups with and without AF, despite methodological adjustments, could potentially introduce bias. The relatively small sample of patients with AF in this study may have contributed to insufficient statistical power for the detection of significant results for specific variables, especially those with lower prevalences. Another major limitation of this study is that it only includes unvaccinated patients, from a cohort attended during the first pandemic wave. This requires a cautious interpretation of the findings.

## 5. Conclusions

Our analysis reveals that pre-existing AF is associated with mortality in hospitalized COVID-19 patients in LATAM, independent of other risk factors. This finding aligns with global research but emphasizes the need for region-specific data. Chronic kidney disease and respiratory rate at admission proved to be critical factors associated with mortality, highlighting the importance of evaluating them in early assessments. While our data on corticosteroid use in COVID-19 show inconclusive effects on mortality, they suggest a complex relationship that warrants further exploration. Overall, our study accentuates the intricate interplay of comorbidities in COVID-19 outcomes and the importance of considering regional patient characteristics in future research.

## Figures and Tables

**Table 1:** Baseline demographics and comorbidities.

Variable	*n* = 3260 ^1^	Atrial Fibrillation		*p*-Value ^2^
		No	Yes	
		*n* = 3145 ^1^	*n* = 115 ^1^	
**Demographics**				
Age	61.0 (48, 71)	60 (48, 70)	72 (65, 80)	<0.001
Male	2059 (63.2)	1991 (63.3)	68 (59.1)	0.4
**Comorbidities**				
Overweight/Obesity	1621 (49.7)	1565 (49.8)	56 (48.7)	0.9
Arterial Hypertension	1596 (49)	1515 (48.2)	81 (70.4)	<0.001
Diabetes Mellitus	869 (26.7)	832 (26.5)	37 (32.2)	0.2
Dyslipidemia	451 (13.8)	416 (13.2)	35 (30.4)	<0.001
COPD	270 (8.3)	242 (7.7)	28 (24.3)	<0.001
Coronary Artery Disease	244 (7.5)	217 (6.9)	27 (23.5)	<0.001
Heart Failure	182 (5.6)	132 (4.2)	50 (43.5)	<0.001
Stroke	102 (3.1)	84 (2.7)	18 (15.7)	<0.001
Cardiac Device	55 (1.7)	40 (1.3)	15 (13)	<0.001

^1^ Median (IQR); *n* (%); ^2^ Wilcoxon rank sum test; and Pearson’s Chi-squared test.

**Table 2:** Baseline clinical characteristics.

Variable	* n * = 3260 ^ 1 ^	Atrial Fibrillation		*p*-Value ^2^
		No	Yes	
		* n * = 3145 ^ 1 ^	* n * = 115 ^ 1 ^	
**Baseline Medications**				
ARB II	805 (24.7)	762 (24.2)	43 (37.4)	0.002
ACEi	358 (11)	330 (10.5)	28 (24.3)	<0.001
Sacubitril/Valsartan	12 (0.4)	10 (0.3)	2 (1.7)	0.091
Betablocker	432 (13.3)	348 (11.1)	84 (73)	<0.001
Aldosterone receptor antagonists	101 (3.1)	72 (2.3)	29 (25.2)	<0.001
SGLT-2i	28 (0.9)	25 (0.8)	3 (2.6)	0.12
Anticoagulant	151 (4.6)	75 (2.4)	76 (66.1)	<0.001
Antiplatelet	351 (10.8)	325 (10.3)	26 (22.6)	<0.001
Statin	398 (12.2)	351 (11.2)	47 (40.9)	<0.001
Diuretic	346 (10.6)	294 (9.3)	52 (45.2)	<0.001
Digitalis	26 (0.8)	15 (0.5)	11 (9.6)	<0.001
**Vital Signs at Admission**				
Heart rate, beats per minute, median (IQR)	93 (80, 106)	93 (80, 106)	86.5 (75, 110)	0.2
Systolic blood pressure, mmHg, median (IQR)	125 (112, 140)	125 (112, 140)	122 (108.5, 136.8)	0.052
Diastolic blood pressure, mmHg, median (IQR)	75 (67, 83)	75 (67, 83)	70 (60.2, 80)	0.021
Respiratory rate, beats per minute, median (IQR)	22.0 (19.0, 28.0)	22.0 (19.0, 28.0)	22.0 (20.0, 26.0)	0.8
**In-Hospital Management**				
Corticosteroids	2197 (67.4)	2121 (67.4)	76 (66.1)	>0.9
Anticoagulation	1257 (38.6)	1172 (37.3)	85 (73.9)	<0.001
Inotropic	336 (10.3)	303 (9.6)	33 (28.7)	<0.001
Vasopressor	900 (27.6)	858 (27.3)	42 (36.5)	0.038
Invasive mechanical ventilation	1115 (34.2)	1074 (34.1)	41 (35.7)	0.8
**Outcomes**				
ICU admission	1745 (53.5)	1683 (53.5)	62 (53.9)	>0.9
In-hospital death	831 (25.5)	785 (25)	46 (40)	<0.001
30-Day post-discharge mortality	53 (2.6)	52 (2.6)	1 (1.6)	>0.9

^1^ Median (IQR); *n* (%); ^2^ Wilcoxon rank sum test; Pearson’s Chi-squared test; ACEI: angiotensin-converting enzyme inhibitors, ARB II: angiotensin II receptor blockers, SGLT2i: Sodium-Glucose Co-Transporter 2 Inhibitors, LV: Left Ventricle, bpm: breaths per minute, COPD: Chronic Obstructive Pulmonary Disease, and IQR: interquartile range.

**Table 3:** Echocardiographic findings of patients with pre-existing atrial fibrillation.

Variable	*n* = 51 ^1^
Left ventricular ejection fraction, median (IQR)	45 (36.5, 58.5)
Systolic dysfunction	24 (47)
Right ventricular dysfunction	17 (33)
Pericardial effusion	7 (13.8)
Severe mitral regurgitation	5 (9.8)
Severe aortic regurgitation	2 (3.9)

^1^ Median (IQR); *n* (%)

**Table 4:** Bivariate analysis of in-hospital mortality.

Clinical Feature	OR	CI 95%		*p*-Value ^1^
		inf	sup	
Atrial fibrillation	1.97	1.21	3.21	0.02
Arterial hypertension	1.64	1.01	2.68	0.11
Diabetes mellitus	1.94	1.16	3.22	0.04
Chronic kidney disease	2.75	1.44	5.27	0.01
Coronary artery disease	1.25	0.61	2.48	0.53
Stroke	1.74	0.72	4.07	0.25
Smoking	1.33	0.71	2.42	0.40
Use of beta-blocker	1.71	1.04	2.79	0.07
Respiratory rate at admission > 25 bpm	1.92	1.18	3.11	0.03
In-hospital pulmonary embolism	1.24	0.31	4.20	0.52
In-hospital acute coronary syndrome	3.59	0.21	14.32	0.28
In-hospital use of corticosteroids	1.84	1.09	3.19	0.07
Invasive mechanical ventilation	10.21	5.99	17.84	>0.01

^1^ Likelihood Ratio Test (Wald Test); Bpm: breaths per minute, OR: odds ratio, CI: confidence interval, inf: inferior, and sup: superior.

**Table 5:** Multiple logistic regression model of factors associated with in-hospital mortality.

Clinical Feature	OR Crude	CI 95%		OR Adjusted	CI 95%		*p*-Value ^1^
		inf	sup		inf	sup	
Atrial Fibrillation	1.97	1.21	3.21	1.85	1.10	3.13	0.044
Chronic Kidney Disease	2.75	1.44	5.27	2.44	1.22	4.89	0.030
Respiratory rate at admission > 25 bpm	1.92	1.18	3.11	2.00	1.21	3.33	0.032
In-hospital use of corticosteroids	1.84	1.09	3.19	1.84	1.07	3.25	0.080

^1^ Likelihood Ratio Test (Wald Test); OR: odds ratio, CI: confidence interval, bpm: breaths per minute, inf: inferior, and sup: superior.

## Data Availability

The data that support the findings of this study are available from the corresponding author upon reasonable request.
